# The Differentiation Balance of Bone Marrow Mesenchymal Stem Cells Is Crucial to Hematopoiesis

**DOI:** 10.1155/2018/1540148

**Published:** 2018-04-03

**Authors:** Jiang Wu, Weiwei Zhang, Qian Ran, Yang Xiang, Jiang F. Zhong, Shengwen Calvin Li, Zhongjun Li

**Affiliations:** ^1^Department of Blood Transfusion, Lab of Radiation Biology, The Second Affiliated Hospital, Army Medical University, Chongqing 400037, China; ^2^Division of Periodontology, Diagnostic Sciences & Dental Hygiene, and Division of Biomedical Sciences, Herman Ostrow School of Dentistry, University of Southern California, Los Angeles, CA 90089, USA; ^3^CHOC Children's Hospital Research Institute, University of California, Irvine, 1201 West La Veta Ave, Orange, CA 92868, USA

## Abstract

Bone marrow mesenchymal stem cells (BMSCs), the important component and regulator of bone marrow microenvironment, give rise to hematopoietic-supporting stromal cells and form hematopoietic niches for hematopoietic stem cells (HSCs). However, how BMSC differentiation affects hematopoiesis is poorly understood. In this review, we focus on the role of BMSC differentiation in hematopoiesis. We discussed the role of BMSCs and their progeny in hematopoiesis. We also examine the mechanisms that cause differentiation bias of BMSCs in stress conditions including aging, irradiation, and chemotherapy. Moreover, the differentiation balance of BMSCs is crucial to hematopoiesis. We highlight the negative effects of differentiation bias of BMSCs on hematopoietic recovery after bone marrow transplantation. Keeping the differentiation balance of BMSCs is critical for hematopoietic recovery. This review summarises current understanding about how BMSC differentiation affects hematopoiesis and its potential application in improving hematopoietic recovery after bone marrow transplantation.

## 1. Introduction

A reciprocal relationship between hematopoiesis and adipocyte differentiation in the bone marrow has long been observed in experiments and in the clinic [1]. In the red marrow, the region of active hematopoiesis in the bone marrow (BM), the number of lipid droplets of adipocytes is significantly decreased (compared to yellow marrow). In contrast, in severe myelosuppressive states such as aplastic anemia or after irradiation, when hematopoietic tissues are damaged, adipocytes expand their lipid contents, indicating that a fatty change occurs in the BM [1]. Thus, bone marrow adipocytes were thought to be a “space-filler” in the BM. In 2009, Naveiras et al. demonstrated that bone marrow adipocytes are negative regulators of hematopoiesis and that they inhibit the expansion of hematopoietic cells in vivo and in vitro [2]. In the same year, Sugimura and Li proposed that the balance between osteogenesis and adipogenesis influences hematopoiesis, which was the first time investigators had recognized that the differentiation balance of BMSCs can affect bone hematopoiesis [3]. BMSCs are important components in the bone marrow, and both osteoblastic cells and adipocytes originate from BMSCs [4]. It has been thought that osteogenesis of BMSCs promotes hematopoiesis and that adipocytes are negative regulators of hematopoiesis [5]. Balance between osteogenesis and adipogenesis is therefore crucial to hematopoiesis. However, the precise mechanism is poorly understood. Recently, large progress has been made to understand the relationship between BMSC differentiation and hematopoiesis. Emerging works have revealed the central role of osteogenesis of BMSCs in bone hematopoiesis. In addition, the role of adipogenesis in hematopoiesis has been demonstrated. Many factors, including aging, obesity [6], irradiation [2], and chemotherapy [7], can lead to the differentiation bias of BMSCs. As a result, bone hematopoiesis can be impaired. In this review, we would concentrate on how BMSC differentiation affects bone hematopoiesis and the critical role of adipo-osteogenic balance of BMSCs in hematopoiesis. Understanding the role of BMSCs and their progeny in hematopoiesis is important. It provides potential targets for alleviating the negative effects of differentiation bias of BMSCs on hematopoietic recovery. In addition, understanding the mechanisms and factors that cause differentiation bias is essential. It provides potential targets for rescuing differentiation bias of BMSCs to promote the hematopoietic microenvironment for hematopoietic recovery after bone marrow transplantation.

## 2. Bone Marrow Mesenchymal Stem Cells and the Hematopoietic Microenvironment

Mesenchymal stem cells (MSCs) are a population of adult stem cells. Although they were first found in the bone marrow and were therefore once termed “marrow stromal cells,” they have since been identified in many tissues such as the umbilical cord and adipose tissue. In vitro, MSCs have the capacity to differentiate into different tissue lineages, and as a result of this property, they may have important roles in regenerative medicine [8]. In vivo, BMSCs are able to differentiate into fat, cartilage, bone, and most of the stromal cells in the bone marrow, thus playing an important role in maintaining hematopoietic stem cells, regulating the hematopoietic microenvironment, and serving a crucial function in the life-long turnover and growth of bone [4].

### 2.1. Potential Markers of BMSCs

In vitro, BMSCs were shown to support long-term hematopoiesis [9]. In vivo, transplants of bone marrow stromal cells into a heterotopic site resulted in bone formation and local hematopoiesis [10]. These early studies indicated the important hematopoietic support capacity of BMSCs. However, there was no specific marker that can be used to identify them in that time. As a result, mechanisms on how BMSCs regulate hematopoiesis were poorly understood. Luckily, several markers, including CD146 in human [11], CXCL12 [12], Nestin [13], leptin receptor [14], and Prx-1 [15] in mice, were shown to be markers of BMSCs. BM cells with each of these markers have the proposed characteristics of BMSCs. They are able to give rise to osteoblast cells and express factors and cytokines that support HSCs. Theoretically, these markers that identify BMSCs in vivo make it possible to study the mechanisms on how BMSCs regulate hematopoiesis. Using some of these markers, BMSCs were shown to predominantly localize around blood vessels and sinusoids in the BM [11, 13].

### 2.2. Hematopoietic Regulation by BMSCs

Using kinds of transgenic mice, BMSCs turn out to be critical for the maintenance of HSCs [16]. BMSCs are the major source of SCF and CXCL12, which is critical for maintenance of HSCs [17]. Deletion of CXCL12-abundant reticular cells (CAR) not only significantly decreased the amount of SCF and CXCL12 but also deleted the HSC in the BM [17]. BMSCs also express Nestin-GFP^+^ transgene, and ablation of Nestin-GFP^+^ BM cells deleted the HSCs in the BM and increased HSCs in the spleen [13]. Moreover, conditional deletion of SCF from LepR-Cre stromal cells [14] or conditional deletion of CXCL12 from Prx-1-Cre cells was also shown to eliminate HSCs in the BM [18]. These emerging studies suggested that BMSCs serve as important niche component supporting HSCs.

Within the total cell population of BMSCs, a distinct subgroup of BMSCs have different functions in regulating HSCs [19]. A small subgroup of BMSCs (NG2^+^ LepR^−^ cells), which belong to small arterioles close to the endosteal region, was shown to contribute to arteriolar niches maintaining HSC quiescence. The other part of BMSCs (NG2^−^ LepR^+^ cells), which are adjacent to sinusoids, is thought to form perisinusoidal niches proliferating HSCs in the BM. Activation of HSC cell cycle changed the distribution of HSCs from these two kinds of niches. Also, conditional depletion of cell expressing NG2 induced HSC cycling [19]. These data revealed the important role of BMSCs themselves in BM hematopoiesis.

In addition, it is believed that the marrow stromal cells generated by BMSCs are crucial to bone hematopoiesis [20]. Marrow stromal cells, including osteoblasts, adipocytes derived from BMSCs, are thought to form the hematopoietic microenvironment [21]. The differentiation of BMSCs, especially to osteogenic and adipogenic lineages, is crucial to bone hematopoiesis.

## 3. Osteogenic Differentiation of Bone Marrow Mesenchymal Stem Cells and Hematopoiesis

At different stages of development, the progenitors that contribute to bone formation are very different. During the embryonic stage of a mouse, the nascent bone tissue derives from Osterix+ progenitors [22]. Given that bone anlagen derives from different germ layers [23], it is proposed that these progenitors have more than one developmental origin, including mesoderm [23] and ectomesenchyme of the neural crest [24]. However, during the adult life of a mouse, LepR^+^ BMSCs are the major source of the bone [25]. In this review, we mainly discuss the BMSCs in adult.

Adult BMSCs can differentiate into many cell types such as adipocytes, osteoblasts, and chondrocytes in the bone. Many methods mimicking these processes in vitro have been developed. For example, osteogenic differentiation can be induced in vitro by bone morphogenetic proteins (BMPs) [26] or by a differentiation cocktail [27]. It has been recognized that Runt-related transcription factor 2 (Runx2) is the key transcription factor in osteogenic differentiation, as activation of Runx2 is considered an initiating event in osteogenic commitment of MSCs [28]. Runx2 can interact with the cis-element on the osteocalcin gene and cause the transcription of several osteoblast-specific genes, inducing osteogenesis in vivo and in vitro [29].

**Figure 1:**
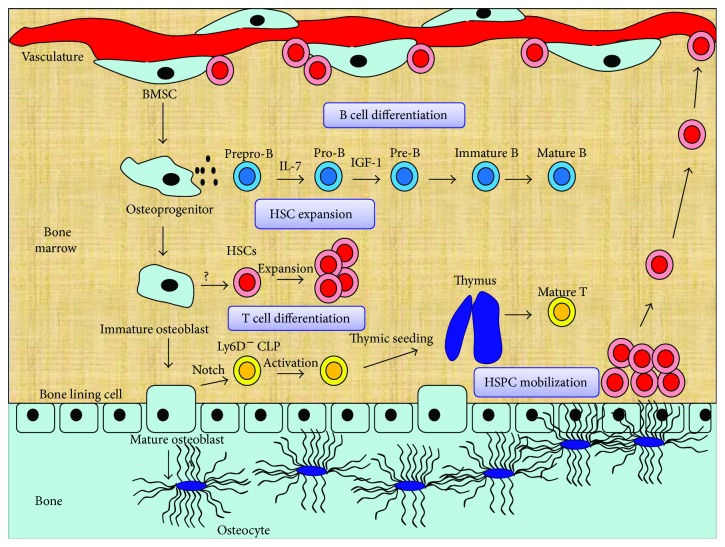
Osteogenesis and hematopoiesis. Distinct cell types in different stages of osteogenic differentiation form unique niches for hematopoietic cells. Osteoprogenitors secrete IL-7 and IGF-1 to support early-stage B lineage differentiation. Osteoblasts are indispensable for HSC maintenance. Immature osteoblasts participate in HSC expansion. Mature osteoblasts express DLL4, which binds to Notch receptor on T cell-competent common lymphoid progenitors (CLP) and induces thymic seeding. Osteocytes conduct lymphoid-supporting stroma and regulate HSPC mobilization.

Osteoblast lineage cells are thought to promote hematopoiesis. In an early study, osteoblasts were shown to support the expansion of hematopoietic progenitors in vitro [30]. Using transgenic mice, Calvi et al. demonstrated the positive correlation between osteoblast lineage cells and HSC number [31]. Conversely, ablation of osteoblast lineage cells results in a severe decrease in the number of HSCs [32]. These results indicate that osteoblast lineage cells can increase the number of HSCs, thus influencing hematopoiesis. Further studies have established the central role of osteoblast lineage cells in bone hematopoiesis. Distinct cell types in different stages of osteogenic differentiation form distinct niches for hematopoietic cell (Figure 1). In general, osteoblast lineage cells include osteoprogenitors, osteoblasts, and osteocytes [33].

### 3.1. Osteoprogenitors Support B Cell Differentiation

Osteoprogenitors are indispensable to B cell differentiation. B cells are derived from CLP in the BM. The developing B cells can be divided into several discrete cell population: prepro-B, pro-B, pre-B, immature B, and mature B [34]. Early studies have demonstrated that genetic disruption of the osteogenic lineage decreased mature B cells in animals [18, 35], indicating that the osteogenic lineage may participate in the development of B cell. Using transgenic mouse, Yu et al. demonstrated that Osx^+^ osteoprogenitors in the BM are essential for B cell differentiation. In the early stage of B cell differentiation, prepro-B to pro-B transition depends on Osx^+^ osteoprogenitor-derived IL-7 [36]. Specifically, although IL-7 can also be produced by other BM components, Osx^+^ osteoprogenitor-derived IL-7 is indispensable for B cell development. Deletion of Gs*α* in Osx^+^ osteoprogenitors resulted in a block of prepro-B to pro-B transition [35]. However, expression of IL-7 in osteogenic lineage cells turned out to rescue B cell development, indicating a critical role of Osx^+^ osteoprogenitors in B cell development [37].

Meanwhile, pro-B to pre-B transition depends on the Osx^+^ osteoprogenitor-derived IGF-1 [36]. Deletion of Osx^+^ osteoprogenitors blocked not only the prepro-B to pro-B transition but also the pro-B to pre-B transition. The addition of IL-7 rescued the prepro-B to pro-B transition, while the addition of IGF-1 rescued the pro-B to pre-B transition [36]. Furthermore, Osx^+^ osteoprogenitors turned out to regulate B lymphocyte mobilization. Specifically, deletion of PPR in osteoprogenitors led to reduced number of B cell progenitors in the BM [33]. However, BM mature B lymphocytes increased. This was associated with the overexpression of VCAM1 caused by PPR deficiency. Taken together, Osx^+^ osteoprogenitors is indispensable for B cell differentiation. The deficiency of Osx^+^ osteoprogenitor could impair B cell maturation and adaptive immune response [33]. PTH signaling in osteoprogenitors, instead of osteoblasts and osteocytes, is indispensable for B cell differentiation [33].

### 3.2. Osteoblast Supports T Cell Differentiation and HSC Function

The process of T cell differentiation includes two stages: prethymic stage and thymic stage [38]. First, BM HSCs give rise to thymus-seeding progenitor of T cells, which migrate to thymus. Second, thymus-seeding progenitors enter thymus and differentiate to naive T cells. After HSCT, the prethymic stage is critical for hematopoietic recovery because the number of thymus-seeding cells is a limit factor for T cell recovery [39].

In prethymic stage, mature osteoblast is indispensable for the production of thymus-seeding progenitors of T cell. DLL4, a Notch ligand expressed in mature osteoblast, is essential for the production of this cell. Deletion of OCN^+^ mature osteoblast decreased common lymphoid progenitors (CLPs) with T cell potential in the BM and downstream T progenitors in the thymus. Also, conditional deletion of DLL4 in OCN^+^ mature osteoblast resulted in similar changes. And recombinant DLL4 was shown to rescue T cell development in OCN^+^ cell-deleted animals.

These data indicate that OCN^+^ mature osteoblasts in the BM provide important molecule for T cell development [40].

Moreover, it was reported that osteoblasts may have a role in HSC function. In early studies, the positive correlation between osteoblast lineage cells and HSC number was demonstrated [31]. Ablation of osteoblast cells led to deletion of HSCs in the BM [32]. Although the methods used to manipulate osteoblast lineage cells were not specific, these initial studies suggested that osteoblasts may have a role in HSC regulation. But this idea is controversial.

In osteoblast lineage cells, mature osteoblast appears to be no longer a direct regulator of HSC. Deletion of OCN^+^ mature osteoblasts, while impairing the generation of T progenitors, did not affect the HSC number in the BM [40]. Also, the expansion of mature osteoblasts turned to even reduce HSC number in the BM [41]. These data indicated that mature osteoblasts may be not a direct regulator of HSC. In another review, Morrison and Scadden believed that mature osteoblasts are essential to HSC function but not necessary to regulate HSCs [42].

Despite the fact that these studies [40, 41] excluded the direct role of mature osteoblasts in HSC regulation, they could not rule out the role of other osteoblast lineage component in HSC regulation. OCN reporter used in these studies could target mature osteoblast but not immature osteoblast [43]. The expression of OCN in immature osteoblasts was shown to be low [44]. Recent studies have demonstrated that hematopoiesis-enhancing activity (HEA) of osteoblast correlated with the expression of Runx2 and osteoblast maturation [44]. Immature osteoblasts express high level of Runx2 and seem to mediate HEA. These cells express CD166, which declines with maturation of osteoblasts and correlates with high HEA [45]. In CD166−/− mice, LT-HSC engrafting was significantly impaired under stress condition, suggesting that immature osteoblasts may regulate HSCs via CD166, and CD166 can be a target to enhance HSC function [46]. Taken together, the immature osteoblast may play a role in HSC expansion and maintenance. However, this population still remains incompletely defined. Developing specific marker that targets immature osteoblasts is necessary for further studies.

### 3.3. Osteocytes Are Indispensable for Lymphopoiesis and HSC Mobilization

Over the decades, the function of osteocytes has been widely studied. Osteocytes account for more than 90% of the bone cells. They are essential for bone health and nonbone organs [47]. Although most of the osteocytes are embedded in the bone, they were found to regulate hematopoiesis. In osteocyte-deleted mice, both B and T lymphopoiesis were severely impaired. Deletion of osteocytes did not affect the BM cellularity, but the early stage of B cell development was blocked. B cell progenitors, including pro-B, pre-B, and immature B, were greatly reduced in osteocyte-less mice. Lack of lymphoid-supporting stroma in the BM was proposed to be the cause of B lymphopenia [48]. In comparison, the thymic atrophy was considered as the major cause of T lymphopenia in osteocyte-less mice. The thymus of osteocyte-less mice failed to support T cell differentiation in vivo. These data indicate that osteocytes are required for lymphoid-supporting stroma in the BM and thymus [48]. However, the precise mechanism is still largely unknown. Osteocytes also regulate HSPC mobilization. In the clinic, G-CSF is used to mobilize HSPCs. However, in osteocyte-less mice, G-CSF failed to mobilize HSPCs into circulation, indicating that osteocytes have an essential role in G-CSF-induced HSPC mobilization [49].

These studies established the central role of osteoblast lineage cells in bone hematopoiesis. Gaining a deeper insight into the biology of these cells, especially the regulation of their differentiation, behaviour, and survival, is valuable. It may potentially provide a better insight into improving hematopoietic recovery.

## 4. Adipogenic Differentiation of Bone Marrow Mesenchymal Stem Cells and Hematopoiesis

BM adipocytes arise from bone marrow mesenchymal stem cells [25]. In vitro, differentiation into white adipocytes can be induced by the addition of insulin, indomethacin, and dexamethasone to the culture medium [50]. It has been thought that peroxisome proliferator-activated receptor gamma (PPAR*γ*) and CCAAT/enhancer-binding protein alpha (C/EBP*α*) are the key transcription factors in adipogenic differentiation. In adipocytes, PPAR*γ*2 can bind to the 5′-flanking region of the P2 gene, which is important for inducing the expression of adipocyte-specific genes and adipogenic differentiation [51, 52].

**Figure 2:**
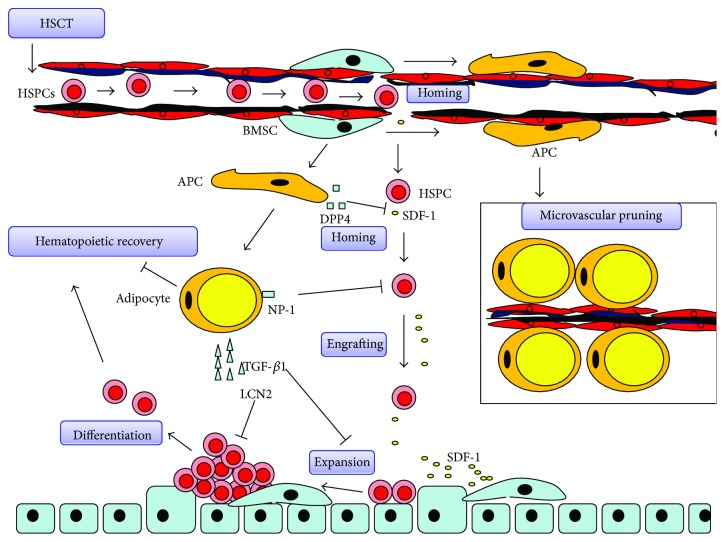
Negative effect of bone marrow adipocytes on hematopoietic recovery. Adipogenic differentiation of bone marrow mesenchymal stem cells interferes the process of hematopoietic recovery after hematopoietic stem cell transplantation (HSCT). Adipocytic lineage, including APC (adipogenic progenitor cell), secretes DPP4 to cleave SDF-1. Moreover, BM adipocyte interacts with HSPCs to downregulate CXCR4 via NP-1, leading to a reduction in SDF-1/CXCR4 signaling and HSPS homing and engrafting. TGF-*β*1 secreted by BM adipocyte mediates cell-cycle arrest of HSPCs to inhibit HSPC expansion. Lipocalin 2 secreted by BM adipocyte inhibits erythropoiesis. DPP4 also cleaves hematopoietic factor including EPO, GM-CSF, G-CSF, and IL-3 to decrease their activity. Furthermore, BM adipocytes replace BMSCs and “pruning” sinusoids, resulting in a reduction of sinus caliber and hematopoietic activity. “Red” marrow then becomes “yellow” marrow in BM.

As a kind of BM stromal cell, BM adipocytes have been thought to be just “space-filler” for many years. When hematopoietic tissues are damaged, adipocytes expand and result in the fatty infiltration in the bone marrow [1]. However, using the transgenic mice, Naveiras et al. first demonstrated that BM adipocytes are negative regulators in BM hematopoiesis under homeostatic and stressed conditions. First, they found that the number of adipocytes correlates inversely with the hematopoietic activity in distinct regions of the mouse BM. After lethal irradiation, the bone marrow space becomes replaced by adipocytes. The transgenic mice, mice without BM fat, showed enhanced hematopoietic recovery postirradiation. Also, pharmacological inhibition of adipogenic differentiation enhanced BM engraftment in wild-type mice [2]. Consistent with that, Lu et al. reported that bone marrow adipogenesis enhanced by rosiglitazone delayed hematopoietic recovery, and hematopoietic recovery [53] is improved by inhibition of adipogenesis following chemotherapy [7]. Those data indicated that BM adipocytes suppress hematopoiesis in hematopoietic microenvironment, at least under stressed conditions. To test this directly, Ambrosi and colleagues transplanted fate-committed adipogenic cells into the tibiae of mice and found a significant reduction in hematopoietic progenitor cells in the BM. Taken together, these data further establish that BM adipocytes significantly attenuate hematopoietic regeneration [6]. Here, we review the negative effect of BM adipocyte on hematopoiesis (Figure 2).

### 4.1. BM Adipocytes Inhibit Hematopoiesis via Cell-to-Cell Contact

In earlier studies, Belaid-Choucair et al. showed that BM adipocytes block granulopoiesis by cell-to-cell contact. Using an antibody-neutralizing neuropilin-1 (NP-1), they restored the granulopoiesis of CD34(+) cells. IL-1*β* and dexamethasone also downregulated NP-1 expression and restored granulopoiesis [54]. In coculture system, adipocytes arrest HSPCs in the G0 phase of the cell cycle and induce apoptosis of HSPCs. CXCR4, an indispensable receptor for HSPC homing and engraftment, is also downregulated on coculture HSPCs. Silencing of NRP1 in adipocytes restored the CXCR4 expression on HSPCs and rescued the apoptosis of them. Those data indicated that BM adipocytes inhibit HSPC homing and engraftment and induce apoptosis of HSPCs via cell-to-cell contact [55].

### 4.2. BM Adipocytes Secrete Cytokines and Factors

Adipocytes differentiated from BMSCs secrete transforming growth factor *β*1 (TGF-*β*1), an inhibitor of hematopoiesis [56]. TGF-*β*1 mediates cell-cycle arrest of hematopoietic cells by upregulating p57KIP2 [57]. In vitro, inhibition of TGF-*β*1 signaling restored expansion of hematopoietic progenitors in coculture system [55]. In vivo, inhibition of TGF-*β*1 signaling accelerates hematopoietic reconstitution after chemotherapy [58] and rescues BM failure in Fanconi anemia [59].

Lipocalin 2 (LCN2), also a secretory protein of adipocyte, has an important function in hematopoiesis. In vitro, LCN2 inhibits erythropoiesis by inducing apoptosis and inhibits differentiation of erythroid progenitors. In vivo, recombinant LCN2 retards recovery from the anemia in mice being affected by acute anemia [60, 61]. Elevated plasma LCN2 was significantly associated with anemia in patients with systemic inflammation [62].

Dipeptidyl peptidase-4 (DPP4), produced by adipocytic lineage [6], cleaves a wide variety of hematopoietic cytokines and factors, including the chemokine stromal cell-derived factor-1 (SDF-1), granulocyte-macrophage colony-stimulating factor (GM-CSF), G-CSF, interleukin-3 (IL-3), and erythropoietin. DPP4 inhibits HSC homing and engraftment [63] and inhibits hematopoiesis in mice [6]. Inhibition of DPP4 may be a promising strategy to enhance hematopoietic engraftment and regeneration [64, 65].

### 4.3. Accumulation of Adipocytes Reduces Blood Flow and Suppresses Hematopoiesis

Bianco et al. showed that adipogenic differentiation of BMSCs resulted in a reduction of the sinus caliber [66]. This process regulates blood flow and hematopoietic activity in the BM [67]. When BMSCs are replaced by adipocytes, sinusoids may be excluded from the blood flow, because BMSCs are physically associated with the sinusoid wall. This process is thought to be reversible because the endothelial wall remains intact. Thus, adipogenesis in the BM is thought to be a unique kind of microvascular pruning to regulate hematopoiesis [68].

## 5. Differentiation Balance of Mesenchymal Stem Cells and Hematopoiesis

Over decades of study, accumulating evidence has demonstrated the reciprocality between osteogenesis and adipogenesis, which is well documented in [69, 70]. Increased adipogenesis often leads to decreased osteogenesis, vice versa [70]. BMSCs are exquisitely balanced for differentiation commitment.

### 5.1. Differentiation Bias of BMSCs Exists in Various Stress Conditions

Many factors, including aging, obesity [6], irradiation [2], and chemotherapy [7], can lead to the differentiation bias of BMSCs. Aging, irradiation, and chemotherapy are known to cause bone loss and marrow adiposity. In aging, microenvironmental alterations are predominately responsible for BMSC lineage switch [71]. Critical microenvironmental signaling, including transforming growth factor-*β* (TGF-*β*), bone morphogenetic protein (BMP), insulin-like growth factor-1 (IGF-1), and Wnt signaling, is altered in aging. In aged mice, TGF-*β* and BMP signaling were shown to be altered correlating with reduced osteogenic differentiation and increased adipogenic differentiation [72]. Also, Wnt3a and Wnt10b, the Wnt ligands that control osteogenic differentiation, significantly decreased with age [73]. IGF-1, the growth factor for osteogenesis, was shown to decline with aging [74]. These microenvironmental alterations may result in age-related bone loss and marrow adiposity. However, the mechanism involved in microenvironmental alterations remains unknown.

The microenvironmental alterations were also observed in radiation injury. In response to radiation, the marrow cells, especially T cell, secreted BMP4 to commit BMSCs to adipogenic lineage [75]. However, the expression of Wnt ligand in BMSCs, was shown to decrease after radiation [76]. Xu et al. also demonstrated that dysregulated lymphocytes may be responsible for the dysregulation of BMSC differentiation and subsequent systemic bone loss after local irradiation [77]. These microenvironmental alterations contributed to the shift of adipo-osteogenic balance of BMSCs.

**Figure 3:**
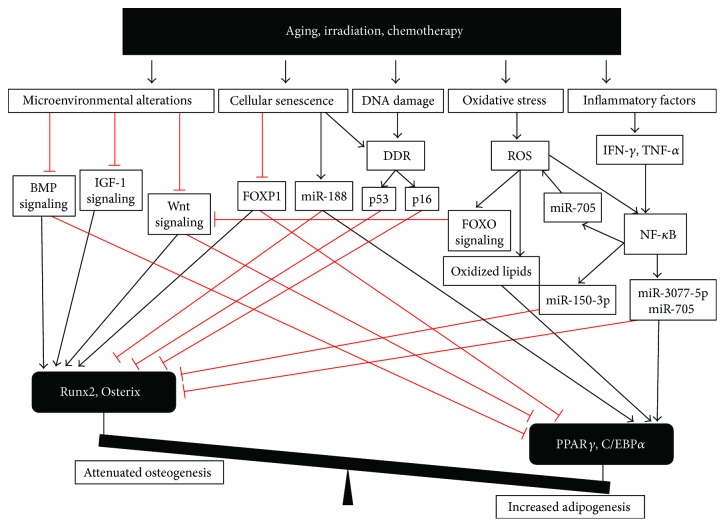
Stress condition-related pathways directly interfere with the differentiation of BMSCs. The differentiation of BMSCs is controlled by a complex signaling network. Runx2 and Osterix are key transcription factors in osteogenic differentiation. And PPAR*γ* and C/EBP*α* are key transcription factors in adipogenic differentiation. However, aging, irradiation, and chemotherapy could cause a series of events and activate subsequent pathways. These pathways regulate key transcription factors Runx2 and PPAR*γ* to lead to the shift of adipo-osteogenic balance.

In addition, other studies also reported that stress condition-related pathways participated in the shift of adipo-osteogenic balance of BMSCs. Aging, irradiation, and chemotherapy could cause a series of events, including oxidative stress, which activates FOXO signaling [78] and PPAR*γ* [79]; DNA damage, which activates p53 and p16 pathway [80–84]; inflammatory factors, which activate nuclear factor *κ*B (NF-*κ*B) pathway [85–87]; and cellular senescence, which leads to alteration of senescent-related molecules involved in differentiation of BMSCs [84, 88]. Unfortunately, most of these activated pathways were shown to directly regulate the differentiation of BMSCs and led to the shift of adipo-osteogenic balance of BMSCs (Figure 3), especially excessive adipogenesis and attenuated osteogenesis.

Cellular senescence results in the decline of FOXP1 [84] and an increase in miR-188 [88], which correlate with reduced osteogenic differentiation and increased adipogenic differentiation.

Senescent cells also showed persistent DNA damage and telomere dysfunction, which lead to DNA damage response (DDR) and subsequent activation of the p53 and p16 pathway [80, 81]. p53 was shown to repress key osteogenic transcriptional factor Osx [82] and inhibit osteogenesis both in vitro and in vivo [83]. p16 was shown to prevent the phosphorylation of pRb, the direct transcriptional coactivator of Runx2. And loss of p16 partially rescued the osteogenesis of senescent BMSCs [84].

Under oxidative stress, FOXO signaling is activated to repress the Wnt signaling and osteogenesis [78]. In addition, oxidized lipids induced by ROS were shown to activate PPAR*γ* to suppress osteoblast differentiation and stimulate adipogenesis [79].

Inflammatory factors [89–91], including TNF-*α* and IFN-*γ*, synergistically impaired osteogenic differentiation in BMSCs via the NF-*κ*B pathway [85]. Further downstream, miR-150-3p [87], miR-3077-5p, and miR-705 [86] were upregulated to mediate the switch from osteogenesis to adipogenesis in BMSCs. TNF-*α* also activated the NF-*κ*B pathway to induce oxidative stress in BMSC via miR-705 and in turn accumulated ROS activated the NF-*κ*B pathway, entering a positive feedback [92]. These pathways regulate key transcription factors Runx2 and PPAR*γ* to control the adipo-osteogenic balance of BMSCs.

### 5.2. Differentiation Bias of BMSCs Interferes with Bone Hematopoiesis

**Figure 4:**
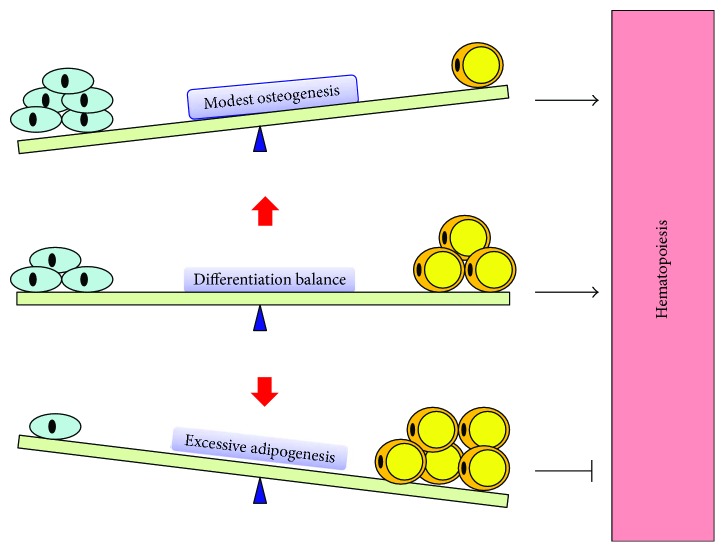
Differentiation balance of mesenchymal stem cells and hematopoiesis. There is a reciprocality between osteogenesis and adipogenesis. Increased adipogenesis often leads to decreased osteogenesis, vice versa. BMSCs are exquisitely balanced for differentiation commitment. Modest osteogenesis of BMSCs promotes hematopoiesis. However, excessive adipogenesis impairs hematopoiesis. Keeping the adipo-osteogenic balance of BMSCs is therefore essential to bone hematopoiesis.

Given the critical role of BMSCs and their progeny in bone hematopoiesis, the differentiation bias of BMSCs could have the inevitable consequence for bone hematopoiesis (Figure 4). Excessive adipogenesis and attenuated osteogenesis inhibit bone hematopoietic recovery. Accumulation of BM adipocytes suppressed hematopoietic recovery in various condition, including aging, obesity [6], irradiation [2], and chemotherapy [7]. Both deletion of BM adipocyte and inhibition of BM adipocyte formation promoted hematopoietic recovery [2]. Meanwhile, attenuated osteogenesis is associated with hematopoietic decline; promoting osteogenesis of BMSCs was also shown to improve hematopoiesis in irradiation [31] and chemotherapy [93].

Of note, although osteogenesis was shown to promote hematopoiesis, excessive osteogenesis may impair hematopoiesis. In an early study, Calvi et al. demonstrated the positive correlation between osteoblast lineage cells and HSC number [31]. However, further study indicated that osteoblastic expansion alone is not sufficient to increase HSC number [41]. In several studies, excessive osteogenesis even inhibited hematopoiesis. In a transgenic mice model, activated Gs signaling in osteoblastic lineage cell leads to a massive increase of bone formation. However, the HSC function and blood production were impaired [94]. Also, Cock et al. reported that the absence of PPAR*γ* in mice led to excessive osteogenesis and reduced BM hematopoiesis [95]. Therefore, although osteogenesis plays a central role in bone hematopoiesis, excessive osteogenesis may impair hematopoiesis. The mode of activation of osteogenesis is critical.

Interestingly, although excessive adipogenesis was shown to inhibit hematopoiesis recovery under stress condition, adipogenesis does not always inhibit hematopoiesis. In contrast, increased hematopoiesis and adipogenesis were observed in several studies. For example, diet-induced obesity results in an increase in adipocytes in mouse BM, and hematopoiesis is enhanced at the same time [96]. What is more, both hematopoiesis and adipogenesis are enhanced by the disruption of TGF-*β* signaling in Smad3-deficient mice [97]. BM adipose tissue also acts as an endocrine organ. Adiponectin secreted by BM adipose tissue enhanced HSC activation [98]. Most recently, Zhou et al. had found that BM adipocyte-derived SCF promoted hematopoietic regeneration after irradiation. And the deletion of SCF in BM adipocytes delayed the hematopoietic recovery of mice [99]. These data indicated that BM adipocytes also produce molecules that benefit hematopoiesis and that BM adipocytes have both negative and positive effect on hematopoietic regeneration. But on the whole, the sum of these effects inhibits hematopoietic recovery under stress condition [2, 6, 7, 53].

At present, it is still unclear why BM fat has a dual effect on hematopoiesis. Recently, emerging work has demonstrated the heterogeneity of BM adipocytes [100]. In different regions of the BM, BM adipocytes have region-specific development, regulation, gene expression, lipid composition, and genetic determinants. They can be divided into rMAT (regulated marrow adipose tissues) and cMAT (constitutive marrow adipose tissues) based on their characteristics [100]. The hematopoietic activity is active in the region of rMAT. But in the region of cMAT, the hematopoietic activity is very low [2, 99]. These data suggested the possibility that different BM adipocytes may have a different effect on hematopoiesis, and the heterogeneity of BM adipocytes may lead to the dual effect. Although this idea is attractive, much work is needed to be done. With transgenic mice model [100], this problem could be solved in further studies. On the whole, inhibiting the BM fat formation was shown to promote hematopoietic recovery in animal models [2, 6, 7, 53].

Taken together, although early studies suggested that osteogenesis of BMSCs promotes hematopoiesis and adipogenesis of BMSCs negatively regulates hematopoiesis, recent studies indicated that both excessive adipogenesis and osteogenesis could impair hematopoiesis. Keeping the adipo-osteogenic balance of BMSCs is therefore crucial to bone hematopoiesis. Rescuing the dysregulation of BMSC differentiation could be a promising strategy for hematopoietic recovery in various conditions.

### 5.3. Promoting Hematopoietic Recovery by Rescuing Differentiation Bias of BMSCs

Differentiation biases of BMSCs, especially excessive adipogenesis and attenuated osteogenesis, were shown to inhibit bone hematopoietic recovery [2, 6, 7]. In animal experiments, pharmaceutical interventions that rescue differentiation bias of BMSCs were shown to promote hematopoietic recovery (Table 1).

BADGE, a PPAR*γ* inhibitor, is a strong inhibitor of adipogenesis of BMSCs [101]. This synthetic compound can bind to PPAR*γ*, the key transcription factor in adipogenesis, and inhibit its activities. It was shown to inhibit adipogenesis in vitro an in vivo [2]. After irradiation and BMT, the administration of BADGE significantly inhibited BM fat formation and promoted hematopoietic recovery in mice [2]. After high-dose chemotherapy, BADGE treatment accelerated the recovery of leukocyte count and number of HSCs in mice [7]. Also, BADGE treatment reduced the rosiglitazone-induced BM fat formation and promoted hematopoietic recovery under hematopoietic stress in mice [53]. These studies indicated that inhibiting adipogenesis of BMSCs could be an effective strategy to promote hematopoietic recovery.

Moreover, promoting the osteogenesis of BMSCs was also shown to enhance hematopoietic recovery. Osteogenic differentiation can be induced in vitro by bone morphogenetic proteins (BMPs) [26] or by a differentiation cocktail of ascorbate, dexamethasone, and *β*-glycerophosphate [27]. Osteogenesis-inducing cocktails (OICS) are known to enhance the activity and expression of Runx2, the key transcription factor in osteogenesis, and promote osteogenesis of BMSCs [102]. After lethal irradiation and BMT, the OICS treatment was shown to promote the hematopoietic recovery and survival of mice [103].

PTH is a critical regulator of calcium homeostasis and osteogenesis. Mechanistically, PTH was proposed to enhance the osteogenesis of BMSCs by promoting the phosphorylation of Smad1/5/8, which are key factors in BMP signaling [104].

It was shown to promote osteogenic differentiation of BMSCs in vivo and in vitro. After myeloablative BMT, intraperitoneal injection of rat PTH for 4 weeks significantly improved hematopoietic engrafting and survival of mice [31]. Also, after multiple rounds of chemotherapy, PTH treatment protected hematopoiesis and preserved the HSC pool in mice [93]. These studies indicated that promoting the osteogenesis of BMSCs could also be an effective strategy to promote hematopoietic recovery.

Taken together, rescuing the dysregulation of BMSC differentiation could be a promising strategy for hematopoietic recovery in various conditions.

## 6. Conclusion and Perspectives

Larger progress has been made to understand the relationship between BMSC differentiation and hematopoiesis. Osteogenesis of BMSCs plays a central role in hematopoiesis, while adipogenesis of BMSCs has a negative effect on hematopoietic recovery. The adipo-osteogenic balance of BMSCs is exquisitely balanced. Many factors, including aging, obesity, irradiation, and chemotherapy, can lead to the differentiation bias of BMSCs and related hematopoietic disorder. Rescuing the dysregulation of BMSC differentiation is crucial to bone hematopoietic recovery. However, there are several questions that remain to be answered.

First, what is the key mechanism that couples osteogenesis and HSC expansion? Early studies reported parathyroid hormone receptor signaling in osteoblast is responsible for the increase in HSC number [31] and indicated that increased expression of Jagged-1 and N-cadherin in osteoblasts is associated with HSC expansion [31]. However, further studies failed to support this idea [105, 106]. Although it was reported that the immature osteoblast may play a role in HSC expansion and maintenance [45, 46], the key molecular mechanisms still remain largely unknown.

Second, given that the osteoblast lineage cells are critical for T and B cell development [36], is attenuated osteogenesis a cause for aging- and obesity-related immune decline? Is the inhibition of osteogenesis a barrier for immune recovery after radiation and chemotherapy? Could it be a strategy to improve immune recovery by promoting osteogenesis?

## Figures and Tables

**Table 1:** Promoting hematopoietic recovery by rescuing differentiation bias of BMSCs.

Targets	Model	Animal	Agent	Dose	Route	Duration	Hematopoietic recovery	Reference
PPAR*γ*	Radiation	Mice	BADGE	30 mg/kg/d	Intraperitoneal injection	2 weeks	↑ WBC, ↑ CFU	[2]
PPAR*γ*	Chemotherapy	Mice	BADGE	60 mg/kg/d	Intraperitoneal injection	4 weeks	↑ WBC, ↑ HSCs	[7]
PPAR*γ*	Chemotherapy	Mice	BADGE	60 mg/kg/d	Intraperitoneal injection	2 weeks	↑ WBC, ↑ CMP, ↑ GMP	[53]
Runx2	Radiation	Mice	OICS	—	Intraperitoneal injection	1 week	↑ WBC, ↑ Hb	[103]
BMP signaling	Radiation	Mice	PTH	80 *μ*g/kg/d	Intraperitoneal injection	4 weeks	↑ HSCs, ↑ CFU	[31]
BMP signaling	Chemotherapy	Mice	PTH	80 *μ*g/kg/d	Intraperitoneal injection	2 weeks	↑ HSCs	[93]

## References

[1] Snyder F. (1965). Fatty acid oxidation in irradiated bone marrow cells. *Nature*.

[2] Naveiras O., Nardi V., Wenzel P. L., Hauschka P. V., Fahey F., Daley G. Q. (2009). Bone-marrow adipocytes as negative regulators of the haematopoietic microenvironment. *Nature*.

[3] Sugimura R., Li L. H. (2010). Shifting in balance between osteogenesis and adipogenesis substantially influences hematopoiesis. *Journal of Molecular Cell Biology*.

[4] Bianco P. (2014). "Mesenchymal" stem cells. *Annual Review of Cell and Developmental Biology*.

[5] Bethel M., Chitteti B. R., Srour E. F., Kacena M. A. (2013). The changing balance between osteoblastogenesis and adipogenesis in aging and its impact on hematopoiesis. *Current Osteoporosis Reports*.

[6] Ambrosi T. H., Scialdone A., Graja A. (2017). Adipocyte accumulation in the bone marrow during obesity and aging impairs stem cell-based hematopoietic and bone regeneration. *Cell Stem Cell*.

[7] Zhu R. J., Wu M. Q., Li Z. J., Zhang Y., Liu K. Y. (2013). Hematopoietic recovery following chemotherapy is improved by BADGE-induced inhibition of adipogenesis. *International Journal of Hematology*.

[8] Kobolak J., Dinnyes A., Memic A., Khademhosseini A., Mobasheri A. (2016). Mesenchymal stem cells: identification, phenotypic characterization, biological properties and potential for regenerative medicine through biomaterial micro-engineering of their niche. *Methods*.

[9] Flores-Guzman P., Flores-Figueroa E., Montesinos J. J. (2009). Individual and combined effects of mesenchymal stromal cells and recombinant stimulatory cytokines on the in vitro growth of primitive hematopoietic cells from human umbilical cord blood. *Cytotherapy*.

[10] Friedenstein A. J., Piatetzky S., Petrakova K. V. (1966). Osteogenesis in transplants of bone marrow cells. *Journal of Embryology and Experimental Morphology*.

[11] Sacchetti B., Funari A., Michienzi S. (2008). Self-renewing osteoprogenitors in bone marrow sinusoids can organize a hematopoietic microenvironment. *Cell*.

[12] Sugiyama T., Kohara H., Noda M., Nagasawa T. (2006). Maintenance of the hematopoietic stem cell pool by CXCL12-CXCR4 chemokine signaling in bone marrow stromal cell niches. *Immunity*.

[13] Mendez-Ferrer S., Michurina T. V., Ferraro F. (2010). Mesenchymal and haematopoietic stem cells form a unique bone marrow niche. *Nature*.

[14] Ding L., Saunders T. L., Enikolopov G., Morrison S. J. (2012). Endothelial and perivascular cells maintain haematopoietic stem cells. *Nature*.

[15] Greenbaum A., Hsu Y. M. S., Day R. B. (2013). CXCL12 in early mesenchymal progenitors is required for haematopoietic stem-cell maintenance. *Nature*.

[16] Mendelson A., Frenette P. S. (2014). Hematopoietic stem cell niche maintenance during homeostasis and regeneration. *Nature Medicine*.

[17] Omatsu Y., Sugiyama T., Kohara H. (2010). The essential functions of adipo-osteogenic progenitors as the hematopoietic stem and progenitor cell niche. *Immunity*.

[18] Ding L., Morrison S. J. (2013). Haematopoietic stem cells and early lymphoid progenitors occupy distinct bone marrow niches. *Nature*.

[19] Kunisaki Y., Bruns I., Scheiermann C. (2013). Arteriolar niches maintain haematopoietic stem cell quiescence. *Nature*.

[20] Flores-Guzman P., Fernandez-Sanchez V., Mayani H. (2013). Concise review: ex vivo expansion of cord blood-derived hematopoietic stem and progenitor cells: basic principles, experimental approaches, and impact in regenerative medicine. *Stem Cells Translational Medicine*.

[21] Dazzi F., Ramasamy R., Glennie S., Jones S. P., Roberts I. (2006). The role of mesenchymal stem cells in haemopoiesis. *Blood Reviews*.

[22] Mizoguchi T., Pinho S., Ahmed J. (2014). Osterix marks distinct waves of primitive and definitive stromal progenitors during bone marrow development. *Developmental Cell*.

[23] Berendsen A. D., Olsen B. R. (2015). Bone development. *Bone*.

[24] Santagati F., Rijli F. M. (2003). Cranial neural crest and the building of the vertebrate head. *Nature Reviews Neuroscience*.

[25] Zhou B. O., Yue R., Murphy M. M., Peyer J. G., Morrison S. J. (2014). Leptin-receptor-expressing mesenchymal stromal cells represent the main source of bone formed by adult bone marrow. *Cell Stem Cell*.

[26] Banerjee C., Javed A., Choi J. Y. (2001). Differential regulation of the two principal Runx2/Cbfa1 n-terminal isoforms in response to bone morphogenetic protein-2 during development of the osteoblast phenotype. *Endocrinology*.

[27] Jaiswal N., Haynesworth S. E., Caplan A. I., Bruder S. P. (1997). Osteogenic differentiation of purified, culture-expanded human mesenchymal stem cells in vitro. *Journal of Cellular Biochemistry*.

[28] Otto F., Thornell A. P., Crompton T. (1997). *Cbfa1*, a candidate gene for cleidocranial dysplasia syndrome, is essential for osteoblast differentiation and bone development. *Cell*.

[29] Zheng H., Guo Z., Ma Q., Jia H., Dang G. (2004). Cbfa1/osf2 transduced bone marrow stromal cells facilitate bone formation *in vitro* and *in vivo*. *Calcified Tissue International*.

[30] Taichman R. S., Reilly M. J., Emerson S. G. (1996). Human osteoblasts support human hematopoietic progenitor cells in vitro bone marrow cultures. *Blood*.

[31] Calvi L. M., Adams G. B., Weibrecht K. W. (2003). Osteoblastic cells regulate the haematopoietic stem cell niche. *Nature*.

[32] Visnjic D., Kalajzic Z., Rowe D. W., Katavic V., Lorenzo J., Aguila H. L. (2004). Hematopoiesis is severely altered in mice with an induced osteoblast deficiency. *Blood*.

[33] Panaroni C., Fulzele K., Saini V., Chubb R., Pajevic P. D., Wu J. Y. (2015). PTH signaling in osteoprogenitors is essential for B-lymphocyte differentiation and mobilization. *Journal of Bone and Mineral Research*.

[34] Nagasawa T. (2006). Microenvironmental niches in the bone marrow required for B-cell development. *Nature Reviews Immunology*.

[35] Wu J. Y., Purton L. E., Rodda S. J. (2008). Osteoblastic regulation of B lymphopoiesis is mediated by G_s_*α*-dependent signaling pathways. *Proceedings of the National Academy of Sciences of the United States of America*.

[36] Yu V. W. C., Lymperi S., Oki T. (2016). Distinctive mesenchymal-parenchymal cell pairings govern B cell differentiation in the bone marrow. *Stem Cell Reports*.

[37] Aguila H. L., Mun S. H., Kalinowski J., Adams D. J., Lorenzo J. A., Lee S. K. (2012). Osteoblast-specific overexpression of human interleukin-7 rescues the bone mass phenotype of interleukin-7-deficient female mice. *Journal of Bone and Mineral Research*.

[38] De Barros S. C., Zimmermann V. S., Taylor N. (2013). Concise review: hematopoietic stem cell transplantation: targeting the thymus. *Stem Cells*.

[39] Zlotoff D. A., Zhang S. L., De Obaldia M. E. (2011). Delivery of progenitors to the thymus limits T-lineage reconstitution after bone marrow transplantation. *Blood*.

[40] Yu V. W. C., Saez B., Cook C. (2015). Specific bone cells produce DLL4 to generate thymus-seeding progenitors from bone marrow. *Journal of Experimental Medicine*.

[41] Calvi L. M., Bromberg O., Rhee Y. (2012). Osteoblastic expansion induced by parathyroid hormone receptor signaling in murine osteocytes is not sufficient to increase hematopoietic stem cells. *Blood*.

[42] Morrison S. J., Scadden D. T. (2014). The bone marrow niche for haematopoietic stem cells. *Nature*.

[43] Roeder E., Matthews B. G., Kalajzic I. (2016). Visual reporters for study of the osteoblast lineage. *Bone*.

[44] Chitteti B. R., Cheng Y. H., Streicher D. A. (2010). Osteoblast lineage cells expressing high levels of Runx2 enhance hematopoietic progenitor cell proliferation and function. *Journal of Cellular Biochemistry*.

[45] Chitteti B. R., Cheng Y. H., Kacena M. A., Srour E. F. (2013). Hierarchical organization of osteoblasts reveals the significant role of CD166 in hematopoietic stem cell maintenance and function. *Bone*.

[46] Chitteti B. R., Kobayashi M., Cheng Y. (2014). CD166 regulates human and murine hematopoietic stem cells and the hematopoietic niche. *Blood*.

[47] Bonewald L. F. (2017). The role of the osteocyte in bone and nonbone disease. *Endocrinology and Metabolism Clinics of North America*.

[48] Sato M., Asada N., Kawano Y. (2013). Osteocytes regulate primary lymphoid organs and fat metabolism. *Cell Metabolism*.

[49] Asada N., Katayama Y., Sato M. (2013). Matrix-embedded osteocytes regulate mobilization of hematopoietic stem/progenitor cells. *Cell Stem Cell*.

[50] Pansky A., Roitzheim B., Tobiasch E. (2007). Differentiation potential of adult human mesenchymal stem cells. *Clinical Laboratory*.

[51] Tontonoz P., Hu E., Graves R. A., Budavari A. I., Spiegelman B. M. (1994). mPPAR gamma 2: tissue-specific regulator of an adipocyte enhancer. *Genes & Development*.

[52] Wu Z., Rosen E. D., Brun R. (1999). Cross-regulation of C/EBP*α* and PPAR*γ* controls the transcriptional pathway of adipogenesis and insulin sensitivity. *Molecular Cell*.

[53] Lu W., Wang W., Wang S., Feng Y., Liu K. (2016). Rosiglitazone promotes bone marrow adipogenesis to impair myelopoiesis under stress. *PLoS One*.

[54] Belaid-Choucair Z., Lepelletier Y., Poncin G. (2008). Human bone marrow adipocytes block granulopoiesis through neuropilin-1-induced granulocyte colony-stimulating factor inhibition. *Stem Cells*.

[55] Ghode S. S., Bajaj M. S., Kulkarni R. S., Limaye L. S., Shouche Y. S., Kale V. P. (2017). Neuropilin-1 is an important niche component and exerts context-dependent effects on hematopoietic stem cells. *Stem Cells and Development*.

[56] Sitnicka E., Ruscetti F. W., Priestley G. V., Wolf N. S., Bartelmez S. H. (1996). Transforming growth factor beta 1 directly and reversibly inhibits the initial cell divisions of long-term repopulating hematopoietic stem cells. *Blood*.

[57] Scandura J. M., Boccuni P., Massague J., Nimer S. D. (2004). Transforming growth factor *β*-induced cell cycle arrest of human hematopoietic cells requires p57KIP2 up-regulation. *Proceedings of the National Academy of Sciences of the United States of America*.

[58] Brenet F., Kermani P., Spektor R., Rafii S., Scandura J. M. (2013). TGF*β* restores hematopoietic homeostasis after myelosuppressive chemotherapy. *Journal of Experimental Medicine*.

[59] Zhang H. J., Kozono D. E., O'Connor K. W. (2016). TGF-*β* inhibition rescues hematopoietic stem cell defects and bone marrow failure in Fanconi anemia. *Cell Stem Cell*.

[60] Miharada K., Hiroyama T., Sudo K., Nagasawa T., Nakamura Y. (2005). Lipocalin 2 functions as a negative regulator of red blood cell production in an autocrine fashion. *FASEB Journal*.

[61] Miharada K., Hiroyama T., Sudo K., Danjo I., Nagasawa T., Nakamura Y. (2008). Lipocalin 2-mediated growth suppression is evident in human erythroid and monocyte/macrophage lineage cells. *Journal of Cellular Physiology*.

[62] Choi J. W., Fujii T., Fujii N. (2016). Elevated plasma neutrophil gelatinase-associated lipocalin level as a risk factor for anemia in patients with systemic inflammation. *BioMed Research International*.

[63] Broxmeyer H. E., Hoggatt J., O'Leary H. A. (2012). Dipeptidylpeptidase 4 negatively regulates colony-stimulating factor activity and stress hematopoiesis. *Nature Medicine*.

[64] Farag S. S., Srivastava S., Messina-Graham S. (2013). In vivo DPP-4 inhibition to enhance engraftment of single-unit cord blood transplants in adults with hematological malignancies. *Stem Cells and Development*.

[65] Lund T. C., Boitano A. E., Delaney C. S., Shpall E. J., Wagner J. E. (2015). Advances in umbilical cord blood manipulation-from niche to bedside. *Nature Reviews Clinical Oncology*.

[66] Bianco P., Costantini M., Dearden L. C., Bonucci E. (1988). Alkaline phosphatase positive precursors of adipocytes in the human bone marrow. *British Journal of Haematology*.

[67] Bianco P., Robey P. G. (2015). Skeletal stem cells. *Development*.

[68] Bianco P. (2011). Bone and the hematopoietic niche: a tale of two stem cells. *Blood*.

[69] Rendina-Ruedy E., Rosen C. J. (2017). Bone-fat interaction. *Endocrinology and Metabolism Clinics of North America*.

[70] Chen Q., Shou P., Zheng C. (2016). Fate decision of mesenchymal stem cells: adipocytes or osteoblasts?. *Cell Death and Differentiation*.

[71] Singh L., Brennan T. A., Russell E. (2016). Aging alters bone-fat reciprocity by shifting in vivo mesenchymal precursor cell fate towards an adipogenic lineage. *Bone*.

[72] Moerman E. J., Teng K., Lipschitz D. A., Lecka-Czernik B. (2004). Aging activates adipogenic and suppresses osteogenic programs in mesenchymal marrow stroma/stem cells: the role of PPAR-*γ*2 transcription factor and TGF-*β*/BMP signaling pathways. *Aging Cell*.

[73] Rauner M., Sipos W., Pietschmann P. (2008). Age-dependent Wnt gene expression in bone and during the course of osteoblast differentiation. *Age*.

[74] Xian L. L., Wu X. W., Pang L. J. (2012). Matrix IGF-1 maintains bone mass by activation of mTOR in mesenchymal stem cells. *Nature Medicine*.

[75] Bajaj M. S., Kulkarni R. S., Ghode S. S., Limaye L. S., Kale V. P. (2016). Irradiation-induced secretion of BMP4 by marrow cells causes marrow adipogenesis post-myelosuppression. *Stem Cell Research*.

[76] Islam M. S., Stemig M. E., Takahashi Y., Hui S. K. (2015). Radiation response of mesenchymal stem cells derived from bone marrow and human pluripotent stem cells. *Journal of Radiation Research*.

[77] Xu X., Li R., Zhou Y. (2017). Dysregulated systemic lymphocytes affect the balance of osteogenic/adipogenic differentiation of bone mesenchymal stem cells after local irradiation. *Stem Cell Research & Therapy*.

[78] Iyer S., Ambrogini E., Bartell S. M. (2013). FOXOs attenuate bone formation by suppressing Wnt signaling. *Journal of Clinical Investigation*.

[79] Tintut Y., Demer L. L. (2014). Effects of bioactive lipids and lipoproteins on bone. *Trends in Endocrinology and Metabolism*.

[80] Farr J. N., Fraser D. G., Wang H. (2016). Identification of senescent cells in the bone microenvironment. *Journal of Bone and Mineral Research*.

[81] Nicolay N. H., Perez R. L., Saffrich R., Huber P. E. (2015). Radio-resistant mesenchymal stem cells: mechanisms of resistance and potential implications for the clinic. *Oncotarget*.

[82] Wang X. Y., Kua H. Y., Hu Y. Y. (2006). p53 functions as a negative regulator of osteoblastogenesis, osteoblast-dependent osteoclastogenesis, and bone remodeling. *Journal of Cell Biology*.

[83] Liu W. J., Qi M., Konermann A., Zhang L. Q., Jin F., Jin Y. (2015). The p53/miR-17/Smurf1 pathway mediates skeletal deformities in an age-related model via inhibiting the function of mesenchymal stem cells. *Aging*.

[84] Li H., Liu P., Xu S. (2017). FOXP1 controls mesenchymal stem cell commitment and senescence during skeletal aging. *The Journal of Clinical Investigation*.

[85] Wang L., Zhao Y., Liu Y. (2013). IFN-*γ* and TNF-*α* synergistically induce mesenchymal stem cell impairment and tumorigenesis via NF*κ*B signaling. *Stem Cells*.

[86] Liao L., Yang X., Su X. (2013). Redundant miR-3077-5p and miR-705 mediate the shift of mesenchymal stem cell lineage commitment to adipocyte in osteoporosis bone marrow. *Cell Death & Disease*.

[87] Wang N., Zhou Z., Wu T. (2016). TNF-*α*-induced NF-*κ*B activation upregulates microRNA-150-3p and inhibits osteogenesis of mesenchymal stem cells by targeting *β*-catenin. *Open Biology*.

[88] Li C. J., Cheng P., Liang M. K. (2015). MicroRNA-188 regulates age-related switch between osteoblast and adipocyte differentiation. *Journal of Clinical Investigation*.

[89] Pawelec G., Goldeck D., Derhovanessian E. (2014). Inflammation, ageing and chronic disease. *Current Opinion in Immunology*.

[90] Lorimore S. A., Coates P. J., Scobie G. E., Milne G., Wright E. G. (2001). Inflammatory-type responses after exposure to ionizing radiation in vivo: a mechanism for radiation-induced bystander effects?. *Oncogene*.

[91] Lu H., Zhu S., Qian L. (2012). Activated expression of the chemokine Mig after chemotherapy contributes to chemotherapy-induced bone marrow suppression and lethal toxicity. *Blood*.

[92] Liao L., Su X., Yang X. (2016). TNF-*α* inhibits FoxO1 by upregulating miR-705 to aggravate oxidative damage in bone marrow-derived mesenchymal stem cells during osteoporosis. *Stem Cells*.

[93] Li S., Zou D., Li C. (2015). Targeting stem cell niche can protect hematopoietic stem cells from chemotherapy and G-CSF treatment. *Stem Cell Research & Therapy*.

[94] Schepers K., Hsiao E. C., Garg T., Scott M. J., Passegue E. (2012). Activated G_s_ signaling in osteoblastic cells alters the hematopoietic stem cell niche in mice. *Blood*.

[95] Cock T. A., Back J., Elefteriou F. (2004). Enhanced bone formation in lipodystrophic PPAR*γ*^hyp/hyp^ mice relocates haematopoiesis to the spleen. *EMBO Reports*.

[96] Trottier M. D., Naaz A., Li Y., Fraker P. J. (2012). Enhancement of hematopoiesis and lymphopoiesis in diet-induced obese mice. *Proceedings of the National Academy of Sciences of the United States of America*.

[97] Epperly M. W., Cao S., Goff J. (2005). Increased longevity of hematopoiesis in continuous bone marrow cultures and adipocytogenesis in marrow stromal cells derived from Smad_3_^−/−^ mice. *Experimental Hematology*.

[98] Masamoto Y., Arai S., Sato T. (2017). Adiponectin enhances quiescence exit of murine hematopoietic stem cells and hematopoietic recovery through mTORC1 potentiation. *Stem Cells*.

[99] Zhou B. O., Yu H., Yue R. (2017). Bone marrow adipocytes promote the regeneration of stem cells and haematopoiesis by secreting SCF. *Nature Cell Biology*.

[100] Scheller E. L., Doucette C. R., Learman B. S. (2015). Region-specific variation in the properties of skeletal adipocytes reveals regulated and constitutive marrow adipose tissues. *Nature Communications*.

[101] Wright H. M., Clish C. B., Mikami T. (2000). A synthetic antagonist for the peroxisome proliferator-activated receptor gamma inhibits adipocyte differentiation. *The Journal of Biological Chemistry*.

[102] Langenbach F., Handschel J. (2013). Effects of dexamethasone, ascorbic acid and *β*-glycerophosphate on the osteogenic differentiation of stem cells *in vitro*. *Stem Cell Research & Therapy*.

[103] Sugino N., Miura Y., Yao H. (2016). Early osteoinductive human bone marrow mesenchymal stromal/stem cells support an enhanced hematopoietic cell expansion with altered chemotaxis- and adhesion-related gene expression profiles. *Biochemical and Biophysical Research Communications*.

[104] Yu B., Zhao X. L., Yang C. Z. (2012). Parathyroid hormone induces differentiation of mesenchymal stromal/stem cells by enhancing bone morphogenetic protein signaling. *Journal of Bone and Mineral Research*.

[105] Bromberg O., Frisch B. J., Weber J. M., Porter R. L., Civitelli R., Calvi L. M. (2012). Osteoblastic N-cadherin is not required for microenvironmental support and regulation of hematopoietic stem and progenitor cells. *Blood*.

[106] Mancini S. J. C., Mantei N., Dumortier A., Suter U., MacDonald H. R., Radtke F. (2005). Jagged1-dependent Notch signaling is dispensable for hematopoietic stem cell self-renewal and differentiation. *Blood*.

